# Global pulses of organic carbon burial in deep-sea sediments during glacial maxima

**DOI:** 10.1038/ncomms10796

**Published:** 2016-02-29

**Authors:** Olivier Cartapanis, Daniele Bianchi, Samuel L. Jaccard, Eric D. Galbraith

**Affiliations:** 1 Institute of Geological Sciences and Oeschger Centre for Climate Change Research, University of Bern, 3012 Bern, Switzerland; 2 Department of Earth and Planetary Sciences, McGill University, Montreal, Canada H3A 2A7; 3 School of Oceanography, University of Washington, Seattle, Washington 98105, USA; 4 Department of Atmospheric and Oceanic Sciences, University of California Los Angeles, Los Angeles, California 90095-1565, USA; 5 Institució Catalana de Recerca i Estudis Avancats (ICREA), 08010 Barcelona, Spain; 6 Institut de Ciència i Tecnologia Ambientals and Department of Mathematics, Universitat Autonoma de Barcelona, 08193 Barcelona, Spain

## Abstract

The burial of organic carbon in marine sediments removes carbon dioxide from the ocean–atmosphere pool, provides energy to the deep biosphere, and on geological timescales drives the oxygenation of the atmosphere. Here we quantify natural variations in the burial of organic carbon in deep-sea sediments over the last glacial cycle. Using a new data compilation of hundreds of sediment cores, we show that the accumulation rate of organic carbon in the deep sea was consistently higher (50%) during glacial maxima than during interglacials. The spatial pattern and temporal progression of the changes suggest that enhanced nutrient supply to parts of the surface ocean contributed to the glacial burial pulses, with likely additional contributions from more efficient transfer of organic matter to the deep sea and better preservation of organic matter due to reduced oxygen exposure. These results demonstrate a pronounced climate sensitivity for this global carbon cycle sink.

## Abstract (web summary)

While numerous studies have indicated that carbon export to the deep ocean was greater during glacial periods, quantification is lacking. Here, via analysis of hundreds of sediment cores, the authors show carbon accumulation rate was 50% higher during glacial maxima than during interglacials.

## Graphical abstract

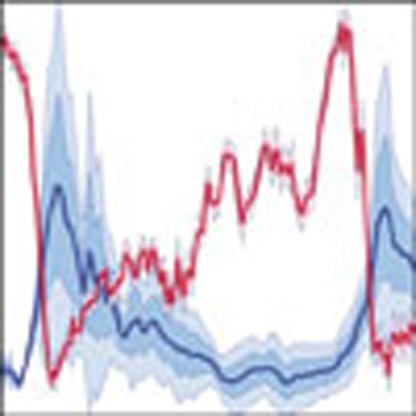

The climate history of the Earth offers a wide range of timescales on which the sensitivity of organic carbon burial to climate might be tested, including the relatively well-documented glacial–interglacial cycles triggered by insolation variations over the quaternary^1^. Much research has focused on changes in the partitioning of carbon between the ocean and atmosphere over these cycles, amplifying orbital forcing by transferring CO_2_ from the atmosphere to the ocean during glacial times, and back to the atmosphere during interglacial periods^1^,^2^,^3^. However, interactions between the long-term storage of organic carbon in oceanic sediments and global climate variations on glacial–interglacial timescales have received little attention.

More than 60 years ago, the discovery of high concentrations of organic carbon in deep-sea sediments near the Galapagos Islands led Arrhenius to propose that exposure of the Galapagos Plateau during the low sea level of the last ice age had caused the resuspension and downslope transport of organic-rich coastal sediments^4^. Later, enhanced burial of organic carbon in glacial-age sediments from the nearby equatorial Pacific was interpreted as reflecting increased biological productivity accompanying intensified wind-driven upwelling^5^, and more recently, reinterpreted as better preservation under reduced oxygenation^6^. Enhanced burial of organic carbon in glacial sediments was also observed in the equatorial Atlantic over several glacial cycles^7^, and at low latitudes for the last glacial cycle^8^, and interpreted as the response of primary productivity to either orbitally forced changes in ocean circulation or to changes in trade winds^7^,^8^.

Here we make a quantitative analysis of several hundreds of high-quality sediment records to extend these pioneering observations to the global scale. We focus our analysis on organic carbon burial in deep-sea sediments, given that the deposition of sediments on continental shelves is complicated by pronounced spatial heterogeneity in biological production and sedimentation (see Methods). Our results suggest that organic carbon burial in deep-sea sediments increased during peak glacial conditions, demonstrating the sensitivity of this component of the global carbon cycle to climatic variation.

## Results

### Organic carbon burial rates during the Last Glacial Maximum

**Figure 1:**
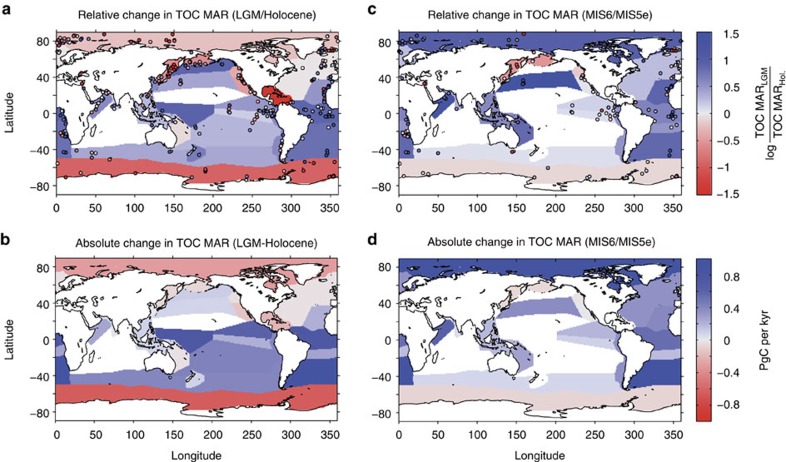
Relative and absolute changes in deep-sea burial of organic carbon for the two last deglacial transition. Relative (**a**,**c**) and absolute (**b**,**d**; PgC per kyr) changes in deep-sea burial of organic carbon from MIS2 to Holocene transition (**a**,**b**) and MIS6 to MIS5e transition (**c**,**d**). Individual sedimentary ratios are shown as coloured circles in **a**,**c**. Shadings correspond to the mean ratio in each province (**a**,**c**) and to the absolute changes in **b**,**d**. Note that (**b**,**d**) show total burial changes in each province, and the absolute changes are not area-normalized.

We estimate global variations of total organic carbon (TOC) mass accumulation rate (MAR) over the past 150 kyr by combining the modern organic carbon burial distribution (see Methods and Supplementary Fig. 1) with time series of TOC MAR from a global compilation of 561 sediment cores extending from the present to the Marine Isotopic Stage 6 (MIS6; Methods). This analysis reveals the existence of robust geographical patterns (Fig. 1a). TOC burial was higher during the Last Glacial Maximum (LGM) in most provinces (Fig. 1a, see Methods and Supplementary Figs 2 and 3), including the tropical and subtropical Atlantic, the eastern and southern Pacific, and the Arabian Sea. The only provinces in which sedimentary TOC burial was lower during the LGM are the high latitudes of the Arctic and Antarctic oceans, the Bering and Okhotsk seas, the California Current, and the Caribbean region. Our reconstruction constrains the global deep-sea TOC MAR during the LGM to between 118 and 171% of the Holocene MAR (Table 1), with a mean estimate of 147±18%. Despite the uncertainties, none of the scenarios that we considered (Table 1, see Methods) allows lower burial during the LGM compared with the Holocene. On the basis of the downcore sediment MAR changes and modern burial map, significantly increased glacial burial occurred in the tropical regions of the Atlantic and eastern Pacific, and the Subantarctic (Fig. 1b). The elevated TOC burial during the LGM is due, in similar proportions, to higher sedimentary organic matter concentrations (126±8% of interglacial value), and to higher sedimentation rates (125±15% of interglacial value, see Methods).

### Organic carbon burial rates during MIS6

A range of factors could potentially bias our LGM/Holocene TOC MAR estimates, including sediment compaction in cores for which density measurements are not available (which would tend to overestimate Holocene MARs) and diagenetic remineralization of TOC (which would tend to underestimate LGM TOC). These biases should be minimized for the penultimate glacial termination, which is generally found at significantly greater depths below the seafloor, where vertical gradients in compaction and labile TOC are far more subdued. Thus, we performed the same analyses for 135 available records in our database that include the MIS6–MIS5e transition (between 135–143 ka and 119–127 ka). Depending on the province map used (Methods), MIS6 burial corresponded to between 148 and 183% of MIS5e TOC MAR, with a mean value of 162±13% (Table 1). Thus, the MIS6/MIS5e ratio shows similar amplitude to the LGM/Holocene ratio. Considering that the climatic conditions during interglacials (MIS5e and the Holocene) were relatively similar, we assume that carbon burial during MIS5e was the same as that during the modern conditions and calculate the global TOC MAR during MIS6 using the MIS6/5e downcore MAR changes (Table 1, Fig. 1c,d). The distribution of changes is very similar to the LGM/Holocene transition, except for the Arctic (Fig. 1c,d.), where age models are arguably poorly constrained^9^.

### Organic carbon burial over the past 150 kyr

**Figure 2:**
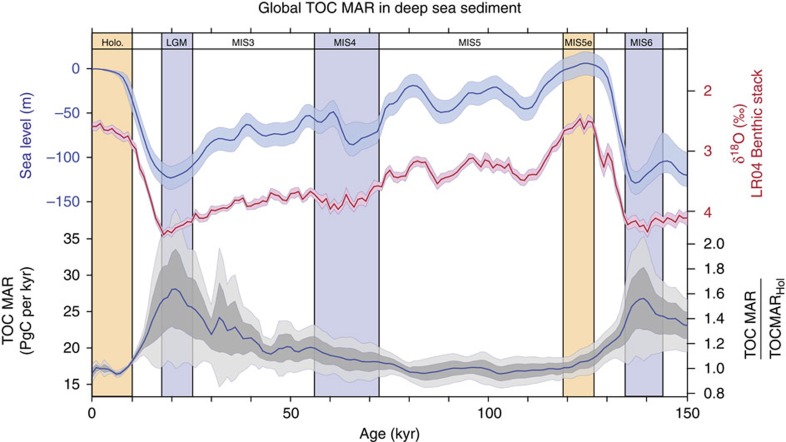
Global TOC burial over the past 150 kyr. Sea-level reconstruction (and associated confidence interval^50^; LR04 benthic foraminifera δ^18^O stack^51^ (±2 s.e.), and reconstructed global organic carbon burial in deep-sea sediment over the past 150 kyr (PgC per kyr,±1σ,±2σ and mean scenario based on the different province scenarios, see Methods, Supplementary Fig. 2 and Supplementary Data 1). The right axis shows relative changes in TOC MAR as compared with Holocene values. The mean organic carbon burial is significantly correlated to both the sea-level reconstruction and the benthic stack (*R*=0.85, *P*<<0.05 and *R*=0.77, *P*<<0.05, respectively). Yellow vertical bands correspond to interglacial periods (Holocene and Marine Isotopic Stage (MIS) 5e), blue vertical bands correspond to glacial condition (Last Glacial Maximum (LGM), MIS4 and MIS6).

Finally, we reconstructed a time series of global organic carbon burial throughout the last glacial cycle by applying the same procedure each 1 kyr time step between 0 and 150 ka (see Methods). The most prominent feature of the reconstructed global organic carbon burial rate over the past 150 kyr (Fig. 2) is the increased burial during glacial maxima, regardless of the province selection strategy (Supplementary Fig. 2). It would therefore appear that the global organic carbon pulses reflect a very consistent response to glacial maxima, which could have resulted from any combination of more rapid export of organic matter from the surface mixed layer, more efficient transfer of organic matter from the upper ocean and continental margins to the seafloor, and better preservation of organic matter in the sediment. We estimate the ‘excess' removal of C from the system that would have occurred during the last glacial period, above the baseline of interglacial C burial rates, by integrating the C burial between 80 and 10 ka, and subtracting the baseline interglacial burial. Our estimate suggests that excess burial in the deep sea, that is, that which would have exceeded a constant interglacial burial flux, removed between 200 and 500 PgC from the ocean–atmosphere system (Table 1).

## Discussion

In general, higher glacial organic carbon burial occurred in regions where a previous qualitative reconstruction, summarizing diverse proxies^10^, inferred higher export production from the surface ocean. The potential importance of increased export is further suggested by similar geographic and temporal patterns between TOC and opal burial (see Methods and Supplementary Fig. 4), which also suggests some degree of increased silicic acid supply to the low latitudes^11^, particularly in the Atlantic. Export production is limited by the supply of nitrogen to the mixed layer over most of the ocean, and by iron and/or other factors limiting growth in nitrate-rich regions^12^. The global nitrate inventory may have been larger during the LGM than at present, due to slower denitrification rates, and a potential fertilization by N_2_ fixing cyanobacteria enhanced by the supply of iron from glacial dust^13^,^14^,^15^. However, isotopic constraints suggest that the fixed N inventory was not >50% and likely not >30% larger than present during the LGM^16^,^17^, which makes it unlikely to have been the sole cause of the observed >50% increase in TOC MAR. Despite the possibility of a larger nitrate inventory, reconstructions from the LGM show much less nitrate at the surface of the Southern Ocean and subarctic Pacific^18^, with low TOC MAR in the coldest parts of these regions, while the high TOC MAR in the Subantarctic is consistent with accelerated export due to dust-borne iron inputs in this region^19^,^20^ (see also Supplementary Fig. 5). Thus, increased export production due to higher dust supply could have contributed to the accelerated burial of organic carbon in presently iron-limited regions, drawing down the available nitrate, while expanded summer sea ice cover and reduced vertical nutrient resupply^21^ likely throttled export production in the coldest oceanic realms. Meanwhile, an intensification of wind-driven upwelling could have provided an additional increase of export production by supplying more nutrients to the tropical Atlantic and Pacific^5^. Despite the potential of these multiple nutrient supply mechanisms to explain the glacial burial peak, they cannot obviously explain the lack of a global burial peak during MIS4, when dust flux was high^20^ and many other features of the global climate were similar to MIS2 (Supplementary Fig. 5).

The transfer of organic matter from the sunlit surface and continental margins to the ocean floor could also have varied over glacial cycles. The transfer efficiency of organic detritus through the water column depends on the types of organic particles produced by the phytoplankton community, the abundance and behaviour of the heterotrophic community that feeds on the organic matter, and the presence of ballast minerals that protect organic matter from bacterial respiration and/or increase sinking velocities of aggregates^22^. Changes in the transfer efficiency could be consistent with the similar spatial and temporal patterns observed in TOC and opal burial (Supplementary Fig. 4). Colder water temperatures may also have increased the transfer efficiency by reducing the metabolic rates of heterotrophs^11^. In addition, both marine and terrestrial organic matter can be transferred from the coastal zone to the deep sea through downslope transport and nepheloid layers. The exposure of the continental shelf during glacial times favoured direct transfer of coastal and riverine sediment to the deep ocean, bypassing temporary storage and partial remineralisation on shelves, and increasing burial efficiencies^23^. Indeed, terrestrial organic carbon deposited at the mouth of the Amazon River under present-day conditions was transferred almost entirely to the deep-sea fan during the LGM^24^,^25^. Increased downslope transport of fine-grained lithogenic material during the glacial sea-level lowstand could also help to explain the increased sedimentation rates evidenced in this study (Methods), and over most major deep-sea fan systems^26^. However, the glacial burial peaks do not appear to have been amplified closer to coasts, as might be expected were downslope transport of shelf sediments the main driver of the global burial pulses (see Methods, and Supplementary Figs 6, 7 and 8).

The third candidate explanation involves enhanced preservation of organic matter in glacial sediment. Increased transport of fine-grained material to the deep sea could have favoured organic matter preservation, which is enhanced with increasing surface area of lithogenic sediment^23^. In addition, organic matter preservation on the seafloor is related to oxygen exposure time^27^, a function of sedimentation rates and bottom water oxygenation. Higher bulk accumulation rates during glacial maxima, potentially due to enhanced dust flux, continental erosion by ice sheets and/or erosion of exposed shelves, would have reduced the oxygen exposure time. Furthermore, proxy reconstructions have shown that the global deep ocean was less oxygenated during the LGM^28^,^29^. A postulated decrease in the proportion of well-ventilated North Atlantic Deep water^30^,^31^ could have contributed to reduced oxygenation of the deep Atlantic^28^ (see Supplementary Fig. 5), and enhanced the burial of organic matter during glacial intervals^32^. However, changes in oxygen exposure time cannot explain covariations between opal and TOC burial (see Supplementary Fig. 4), and the non-linear relationship between oxygen exposure time and burial efficiency^27^ suggests that this process may have less leverage in the deep sea, where sedimentation rates are low. It is possible that both enhanced TOC and opal burial reflect better preservation due to increased bulk sedimentation rates^33^,^34^.

The glacial burial pulses documented here, and their correlation with sea level and global ice volume (Fig. 2), are evidence of a striking sensitivity of deep-sea organic carbon burial to climate. The excess burial of 200–500 PgC of organic carbon in deep-sea sediment during glacials is comparable to the excess carbon stored below ice sheets^35^, or within soil, permafrost and peat deposits^36^ during glacials. While our results constrain the deep-sea organic carbon sink during glacials, a closure of the long-term carbon budget in the ocean will require an estimate of deep-sea calcium carbonate burial, as well as burial on shelves. In addition, the fact that a single major deep-sea fan off of the Amazon basin accounts for a significant part of glacial organic matter burial points to the need for studies specifically targeting deep-sea fans and slopes.

Three general factors have been presented here that could have contributed to the burial pulses, acting individually or together. The apparent lack of an organic carbon burial peak during MIS4, an interval in many respects similar to MIS2 and MIS6, is an intriguing observation that deserves further attention. Disentangling the interwoven causes behind the burial pulses provides an important challenge for understanding the global carbon cycle, and its variations through Earth history.

## Methods

### Analytical strategy overview

Our estimate for carbon burial involves three consecutive steps. First, we use a global map of organic carbon burial in modern (core-top) sediments, based on ref. 37 (Supplementary Fig. 1). Second, in order to infer regional changes in burial patterns from individual sediment cores while accounting for the geographic and bathymetric variability in global organic carbon burial, we subdivide the world ocean into different sets of geographical provinces. Given that any such division will introduce inherent biases, we used a total of seven different subdivision strategies (Supplementary Fig. 2), and compare the results, as a test of the degree to which the results depend on the selection of provinces, and to evaluate uncertainties. Third, we use quality-controlled sediment records to reconstruct relative changes, as compared with the Holocene, of the burial rate in each province. These composite time series are then used, in combination with modern estimates of burial rates, to calculate absolute changes in the burial rates in each province.

### Modern TOC MAR and associated uncertainties

The modern organic carbon MAR map (Supplementary Fig. 1) was generated by multiplying the TOC concentrations reported for surface sediments (core tops) by the corresponding bulk sediment accumulation rate. The TOC content map was generated by combining the map reported by ref. 38, augmented with unpublished data, while the MAR map was determined based on the geometric mean of two pre-existing maps (ref. 39 and other unpublished data, see details in ref. 37). It is important to note that both the MAR and the TOC maps have insufficient spatial resolution to adequately resolve continental margins. Even though a substantial portion of organic carbon burial may occur in coastal environments (up to 90% (ref. 40), our maps with a 1 × 1degree spatial resolution cannot resolve small-scale features such as shorelines, shelves and deep-sea fans. Uncertainties related to the modern burial estimates may have two impacts on our results:

(1) The absolute values of the modern burial conditions and the burial values inferred for the past. The modern map that we use is consistent with the most commonly cited value for deep-ocean organic carbon burial^41^, but other studies have reported values that diverge by orders of magnitude (see details and references in ref. 42). For this reason, we reported relative changes in Table 1, based on the downcore records only. Similarly, the right *y* axis on the bottom panel of Fig. 2 shows relative changes as compared with the Holocene, rather than absolute values.

(2) A different spatial distribution of the modern burial would change the relative contribution of the provinces to the global budget, altering both the pattern and absolute value of the reconstructed global TOC burial. To test the impact of a modern map with TOC burial focused on continental slopes and coastal environments, we used the global map of modelled organic carbon flux to the seafloor from ref. 43. This map shows a similar pattern in the deep sea, but orders of magnitude higher flux in coastal regions compared with the map used in our study (note that the flux of TOC to the seafloor differs from burial, because of organic carbon remineralization on the seafloor; Supplementary Fig. 9, blue axis). Following exactly the same approach, but using this alternative map as modern reference, did not substantially modify the shape and amplitude of the relative global TOC burial changes (Supplementary Fig. 9, blue lines), as compared with the reference maps (Supplementary Fig. 9, red lines).

We further tested the influence of the modern burial map on our results, using an updated TOC content map, and only the Jahnke bulk MAR map^39^ (Supplementary Fig. 9, green lines). Once again, the relative changes in global burial obtained were very similar to the one presented in this study, suggesting that our reconstruction is robust and relatively independent on our starting assumptions in terms of variability.

Because paleoceanographic studies favour using sedimentary archives that are continuous, and hence not perturbed by abrupt sedimentary processes such as mass flows or turbidity currents, or by hiatuses in sedimentation rates, the coring sites are generally expected to have been selected to avoid such processes. However, sedimentation over slopes and deep-sea fans probably occurs mostly as massive and sporadic, yet very localized events. Meanwhile, some portions of the ocean floor probably see no sediment deposition at all, or even erosion. These types of sedimentary environments are likely under sampled by the sediment cores used in this study.

Thus, our reconstruction applies mainly for deep-sea sediment, poorly resolving coastal sediment deposits. No MAR map is available for the Mediterranean Sea to our knowledge, and as a consequence this region was not considered further.

### Province definition strategies

The first two strategies subdivide the global ocean into the major ocean basins (Supplementary Fig. 2a), as well as finer subdivisions of the oceans into seas (Supplementary Fig. 2b), based on ref. 44. Next, based on the assumption that the large-scale ocean features driving biogeochemical cycles in the modern ocean have remained relatively stationary over time, we used the annual climatology of the ocean biogeochemical provinces based on ref. 45, later updated by ref. 46. This map defines 56 coherent provinces from a biogeochemical perspective (Supplementary Fig. 2c). To adapt the subdivision of the ocean to our sample distribution, the Longhurst map was redefined and simplified into two different maps with, respectively, 30 (Supplementary Fig. 2d) and 15 different provinces (Supplementary Fig. 2e). To investigate potential influences of the depth of the record, these two simplified maps were used to create two new sets of maps by dividing each province into a shallow and a deep component using the 1,500 m isobaths (Supplementary Fig. 2f,g).

### Extracting burial rate changes from sediment core database

First, we created a database comprising surface and downcore sediment composition, retrieving available data from the NOAA (ftp://ftp.ncdc.noaa.gov/pub/data/paleo/paleocean/sediment_files/) and PANGAEA (http://www.pangaea.de) online repositories (Supplementary Data 2). Thus, any data set used in this study is available online in one of these repositories. All the data sets that contained TOC concentrations, TOC accumulation rates, age models, sediment density values, along other parameters were taken into consideration. Given that some records are reported with their original depths, while others can be reported with composite or corrected depth scales (from drilling disturbance, voids, or instantaneous deposits such as turbidites or tephras), we first verified the internal consistency of the depth scale, by comparing similar proxies from different records from the same core. When more than one combination between TOC content, age model and density was possible, the best one was selected. The objective criterion used to select the best combination was as follows: outliers detected visually from the cloud of different solutions were removed. More recent versions of age models were favoured over older versions. Age models with a high number of tie points and calibrated ^14^C measurements were preferred, and a composite age scale was created for some of the records, using least square splines.

230-Thorium normalized sediment accumulation rates were used whenever available. If they were not available (the majority of records), sedimentation rates were inferred at the depth of each TOC measurement by calculating the linear sedimentation rate implied by the age model. We used measured dry bulk densities, when available, interpolated onto the TOC record sampling depth, to convert linear accumulation rate to MAR. When dry bulk densities were not directly available, we assumed constant values equal to 0.9 g cm^−3^, corresponding to the mean dry bulk density for compacted marine sediments in our database.

Each sedimentary record of organic carbon accumulation was expressed as a ratio to the mean Holocene value of the same record. The mean Holocene to LGM ratio of sedimentary records was calculated for each province (Fig. 1a), and used to estimate LGM TOC burial by multiplying the province ratio with the corresponding province modern burial value (Fig. 1b, Table 1). The LGM global burial rate was then obtained by summing the inferred burial in each province (Table 1). Note that we used the geometric, rather than the arithmetic mean of LGM to Holocene ratios, given that the values are log-normally distributed.

Our database includes 561 total TOC MAR time series. Of these, 260 were of sufficient resolution and length for both the Holocene (defined as 0–10 ka; 454 records), and the LGM (18–25 ka; 303 records) to calculate average LGM to Holocene TOC MAR ratios (coloured circles, Fig. 1a). The provinces for which no downcore records are available account for a small fraction (0 to 23%, depending on the province strategy) of modern global burial. Significant variations of sedimentary LGM/Holocene ratios occur within a single province, indicating local variability in the factors affecting the production, transfer and sedimentation of organic matter (Supplementary Fig. 3).

The same procedure was used to calculate global TOC burial rate over the past 150 kyr with a 1 kyr time step. We used interpolated downcore MAR records with a 1 kyr time step, expressed as a fraction of Holocene MAR (0–10 kyr). Any time interval within a sediment record in which no measurement was present over a period >10 kyr was excluded from the calculation of the regional stacks. When no record was available for a specific province, and/or for a specific period, the province flux was assumed to be constant and equal to modern values.

To evaluate the relative influence of organic carbon content and sedimentation rate variations on the results, we also calculated global burial assuming (1) constant sedimentation rates and (2) constant TOC content. This test indicates that the more elevated TOC burial during the LGM is due, in similar proportions, to higher sedimentary organic matter concentrations (126±8% of interglacial value) and to higher sedimentation rates (125±15% of interglacial value).

Given that the deglacial trend in organic matter burial in the Arctic is uncertain, and shows opposite behaviour from MIS6 to MIS5e, and LGM to Holocene transitions, we excluded the Arctic Ocean. As only a small proportion of global burial currently occurs in the Arctic Ocean (<8%), this exclusion has only a very minor impact on the results in terms of timing and amplitude.

### Long-term trend in global organic matter burial

The mean of the different scenarios shows a pronounced increasing trend towards the present (Supplementary Fig. 10). There are different explanations to account for that trend, which are not related to actual burial changes. Given that density measurements were not always available, we performed our calculation assuming a constant density for the entire core. The consequence of this assumption is that the calculated MARs are slightly overestimated for the most recent sediments, for which the density is expected to be lower than for older sediments, because of sediment compaction. This can also partly explain the increasing trend during the late Holocene (Supplementary Figs 5 and 10). In addition, the slow long-term diagenesis of refractory organic carbon contributes to the gradual decrease of apparent burial fluxes with age.

Another source of uncertainty arises from the use of raw ^14^C ages to derive some sediment core-age models. Records for which only raw ^14^C ages were available should slightly overestimate the most recent calculated TOC MAR, due to the difference between calendar and ^14^C ages. Thus, these records can slightly reduce the LGM to Holocene amplitude, but cannot explain the decreasing trend. This is confirmed by similar patterns between MIS6 and MIS5, out of the range covered by ^14^C-dating.

Finally, apparent sedimentation rates decrease as a power law function of the intervals between two age control^47^, indicating that sedimentation rates could be overestimated during the Holocene and the deglaciation, when the age model resolution is generally higher, as compared with older periods.

To correct for these biases, we removed the long-term trend from each scenario (Supplementary Fig. 10) calculated using a least-squares spline modelling tool (MATLAB Shape Language Modeling toolbox, D' Errico, 2009, MATLAB Central File Exchange, retrieved online on February 2012). It is important to note that this correction implicitly assumes similar burial rates for the Holocene and MIS5.

### Biogenic opal burial variations

To evaluate the changes in opal burial over the past 150 kyr, we applied the same method described in this paper for TOC burial to biogenic opal records available in our database. The number of sedimentary records for opal burial was lower than for TOC. However, we obtained remarkably similar results from a spatial and temporal point of view, in provinces with a sufficient number of records (Supplementary Fig. 4).

### Impact of sea-level change on sediment redistribution

As sea level dropped during glacial periods, continental shelves were exposed and eroded, activating submarine canyons and rerouting coastal deposits directly to the deep sea^48^. Indeed, the present-day deposition of terrestrial organic carbon at the mouth of the Amazon River was transferred almost entirely to the deep-sea fan during the LGM, representing 3.7 PgC per kyr^24^, or >13% of global LGM deep-sea burial rate (Table 1). Although this particular hot spot in marine accumulation rate is not resolved in our analysis, it is possible that downslope transport of organic matter does contribute to some continental slope records included in our database. Despite its importance, this mechanism is unlikely to explain more than a fraction of our reconstructed changes in TOC MAR, given that many of the records in our database are far from continental slopes. It is worth noting that erosion of coastal deposits during sea-level lowstands may have enhanced nutrient availability and productivity of the ocean^48^,^49^, while increased organic matter reaching the seafloor may have contributed to reduced oxygenation of the deep sea^48^.

To further test the potential influence of coastal deposit remobilization during sea-level lowstands, we performed additional analyses, following exactly the same procedure as outline above, using newly designed province maps, for which we distinguished coastal and open ocean regions. Assuming that glacial–interglacial sediment remobilization/relocation was more important near shorelines, we used the ocean and seas maps, as well as the two simplified Longhurst maps (Supplementary Fig. 6) and split each province into a coastal and an open ocean province. Coastal regions were defined using distance thresholds of 500, 1,000 and 1,500 km from the closest point on the coastline (Supplementary Fig. 6). Changes of global TOC burial based on these nine new province maps were similar to the previous analyses, in terms of shape and absolute value (Supplementary Figs 6 and 7). Moreover, the burial in open ocean and in coastal regions displays similar patterns to the whole deep ocean regardless of the width of the coastal provinces, suggesting similar temporal patterns in both open ocean and coastal regions (Supplementary Fig. 7). Finally, we calculated the distance from the shelf, here defined as the 150 m isobath, for each individual sediment core (Supplementary Fig. 8). The absence of correlations between the MAR change between the Holocene and the LGM, and the distance from the shelf, further suggests that regional patterns of burial dominate over continental influences.

## Additional information

**How to cite this article:** Cartapanis, O. *et al.* Global pulses of organic carbon burial in deep-sea sediments during glacial maxima. *Nat. Commun.* 7:10796 doi: 10.1038/ncomms10796 (2016).

## Supplementary Material

Supplementary Information Supplementary Figures 1-10 and Supplementary References

Supplementary Data 1 Global TOC MAR in deep sea sediment for several province map, and mean scenario.

Supplementary Data 2 Total Organic Carbon (TOC) mass accumulation rate (MAR) compilation

## Figures and Tables

**Table 1:** Changes in organic carbon burial over the two last glacial–interglacial transitions.

**Province map**	**Holocene MAR (PgC per kyr)**	**LGM/Holocene (%)**	**LGM MAR (PgC per kyr)**	**MIS6/MIS5e (%)**	**MIS6 MAR (PgC per kyr)**	**Glacial excess burial (PgC)**	**Number of provinces**
Ocean	17.1	117.7	20.1	152.0	26.0	196	7
Seas	17.1	134.7	23.0	153.2	26.2	242	101
Longhurst (L.)	16.8	159.3	26.8	148.2	24.9	338	56
Modified L. 1	17.1	141.0	24.1	155.6	26.6	262	30
Modified L. 1+depth.	17.1	147.0	25.1	167.3	28.6	281	60
Modified L. 2	17.1	158.6	27.1	176.0	30.1	435	15
Modified L. 2+depth.	17.1	170.7	29.2	183.0	31.3	501	30
Mean		147.00	25.06	162.19	27.67	322	
s.d.		17.75	2.99	13.38	2.37	110	

Holocene and LGM deep-ocean TOC burial (MAR) estimated for the different province maps (shown in Supplementary Fig. 2). A subdivision of the provinces following the 1,500 m isobath was added for province maps 5 and 7. The glacial excess burial was calculated between 80ka, when global burial diverged from MIS5 values, and 10ka, when global burial reached low Holocene values (Fig. 1).
