# Supplementary material for: Global pulses of organic carbon burial in deep-sea sediments during glacial maxima
Source: Nat Commun. 2016 Feb 29;7:10796. doi: 10.1038/ncomms10796 (PMC4773493; doi:10.1038/ncomms10796)
Supplement: Supplementary Information — Supplementary Figures 1-10 and Supplementary References [file ncomms10796-s1.pdf]

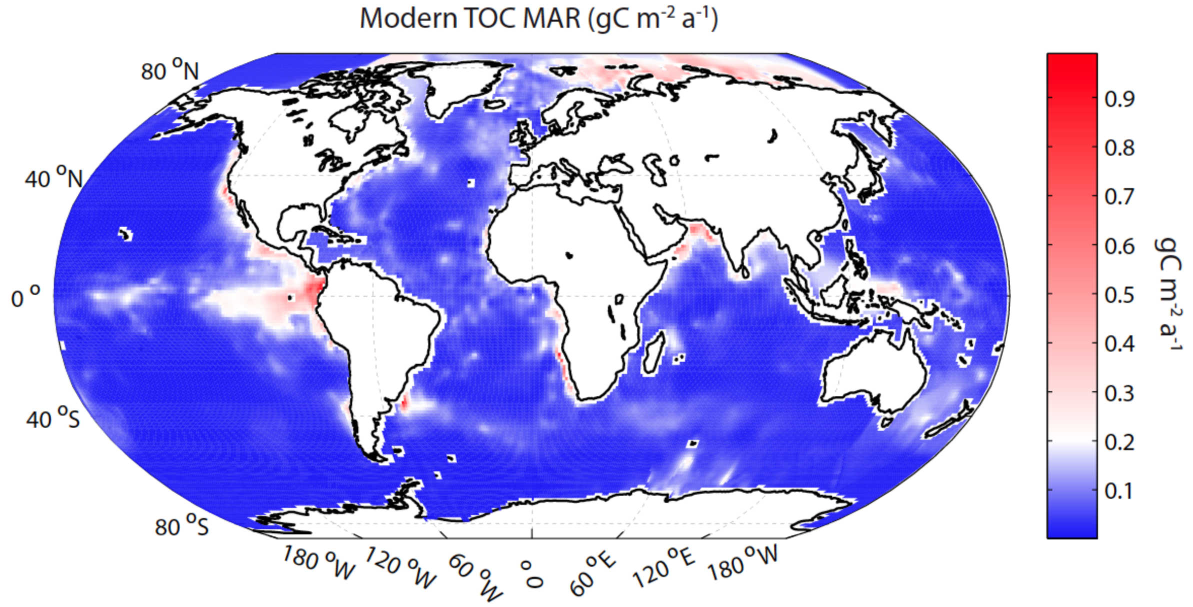

Supplementary Figure 1:
Modern burial of organic carbon in deep sea sediment (gC m^{-2} a^{-1}) [1].

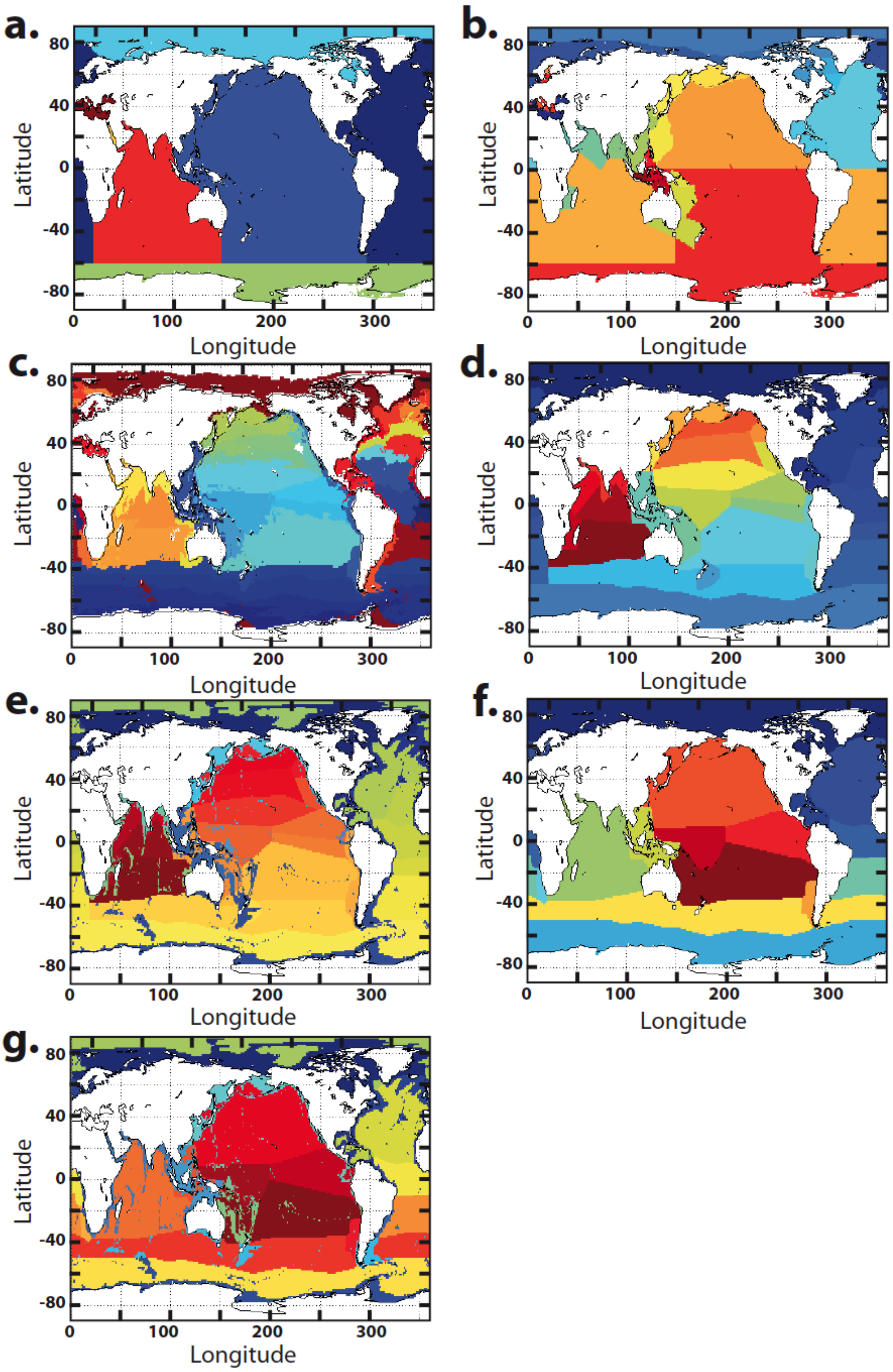

Supplementary Figure 2:
The 7 province maps used in this study. Each color corresponds to a single province: a. Ocean subdivision following [2]; b. Seas following [2]; c. Longhurst Bioregion [3] updated by [4]; d. Simplified Longhurst bioregion 1 (30 provinces); e. Simplified Longhurst bioregion 1 with depth subdivision at 1500 m (60 provinces); f. Simplified Longhurst bioregion 2 (15 provinces; used in Fig. 1); e. Simplified Longhurst bioregion 2 with depth subdivision at 1500 m (30 provinces).

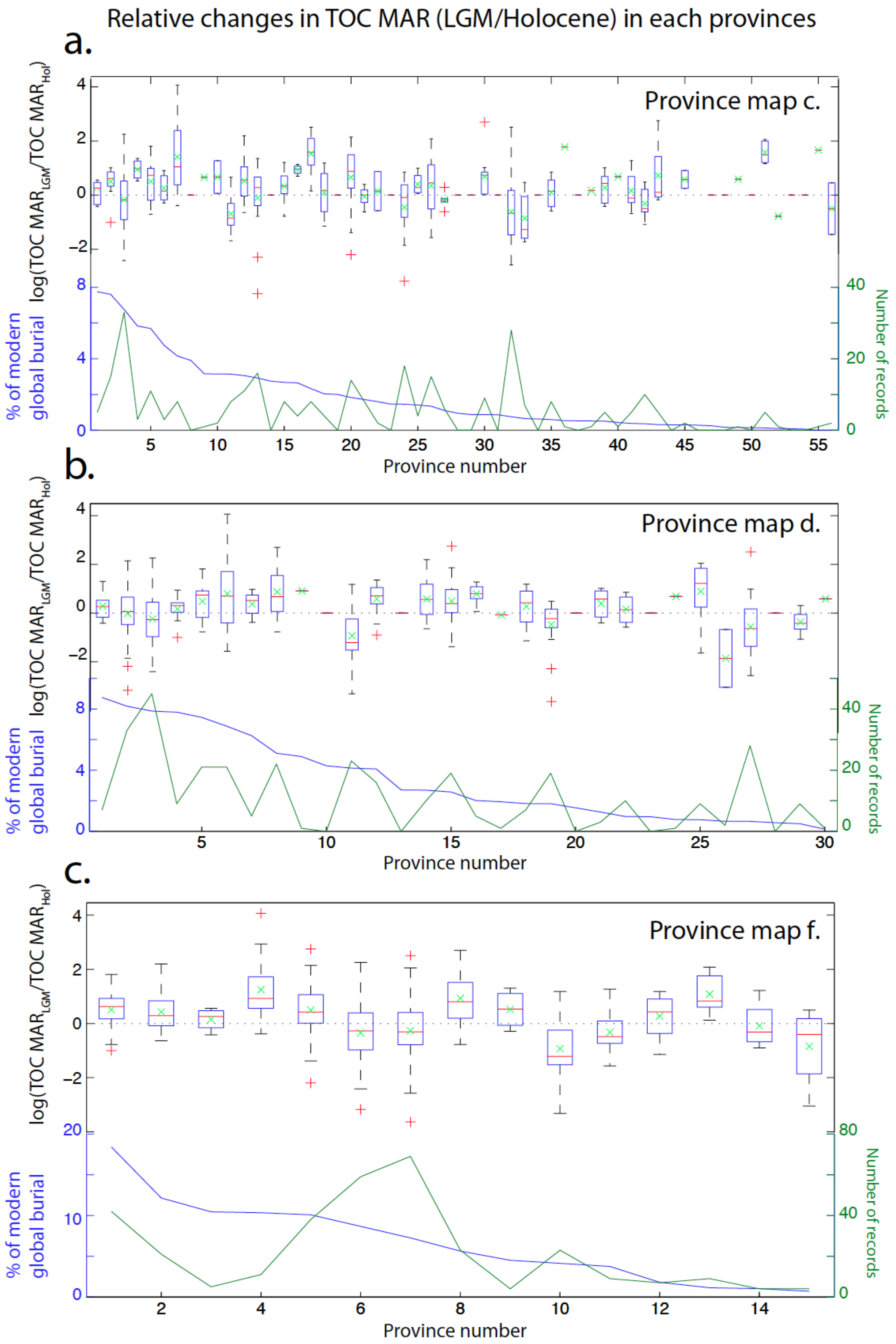

Supplementary Figure 3:
Relative changes in downcore TOC MAR (LGM/Holocene) in each province for province map .c, .d, and .f in Supplementary figure 2 (panel a. , b. , and c. respectively). Panel b. corresponds to the results show in Fig. 1a. Box plot shows 0 % (bottom whiskers), 25 %, 50 % (median; red lines), 75 %, and 100 % (top whiskers) quantiles, as well as mean (green cross) and outliers (red cross), for each provinces. Provinces were sorted depending on their contribution to modern TOC MAR (bottom blue curve), and the number of record used is displayed in green.

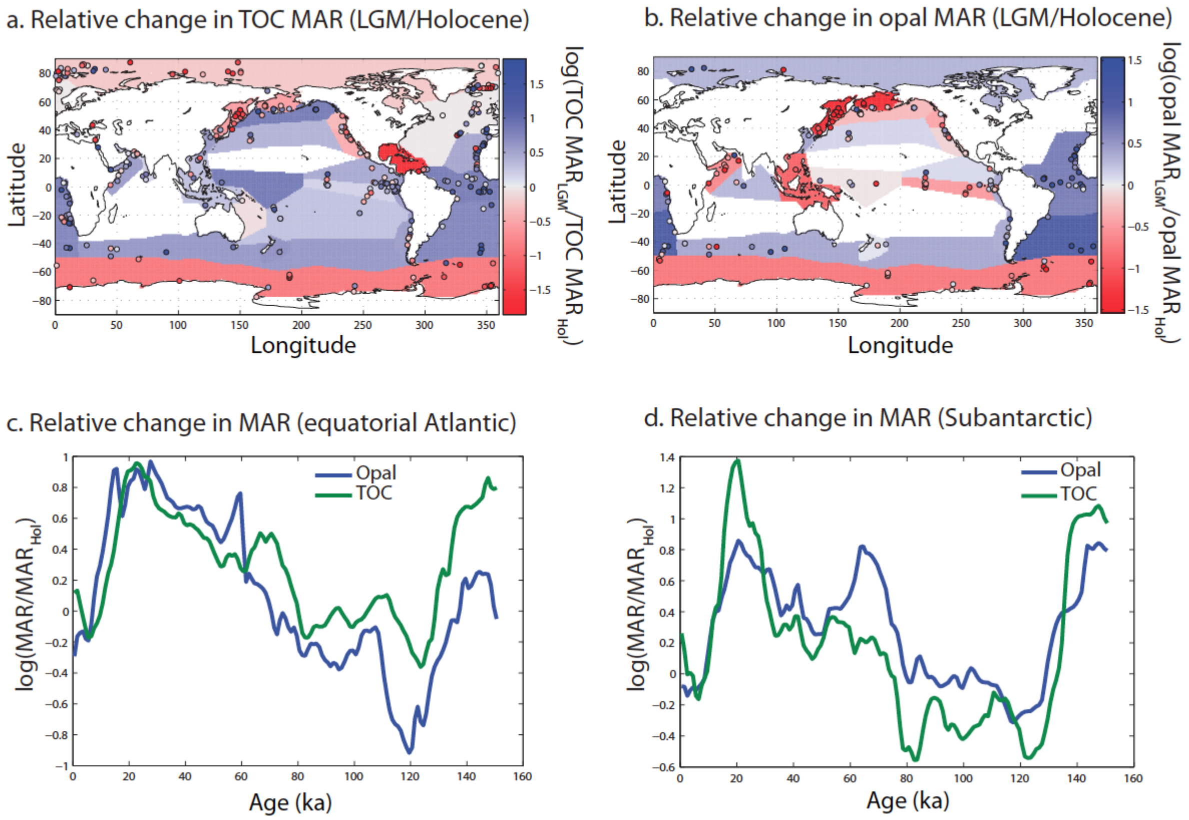

Supplementary Figure 4:

Relative changes in deep-sea TOC MAR (a) and biogenic opal MAR(b) for LGM to Holocene transition. Shadings correspond to the mean ratio in each province (province map d). Relative changes in TOC and opal MAR in equatorial Atlantic (c), and Subantarctic zone (d) over the last 150ka (province map f. in Supplementary Fig. 2).

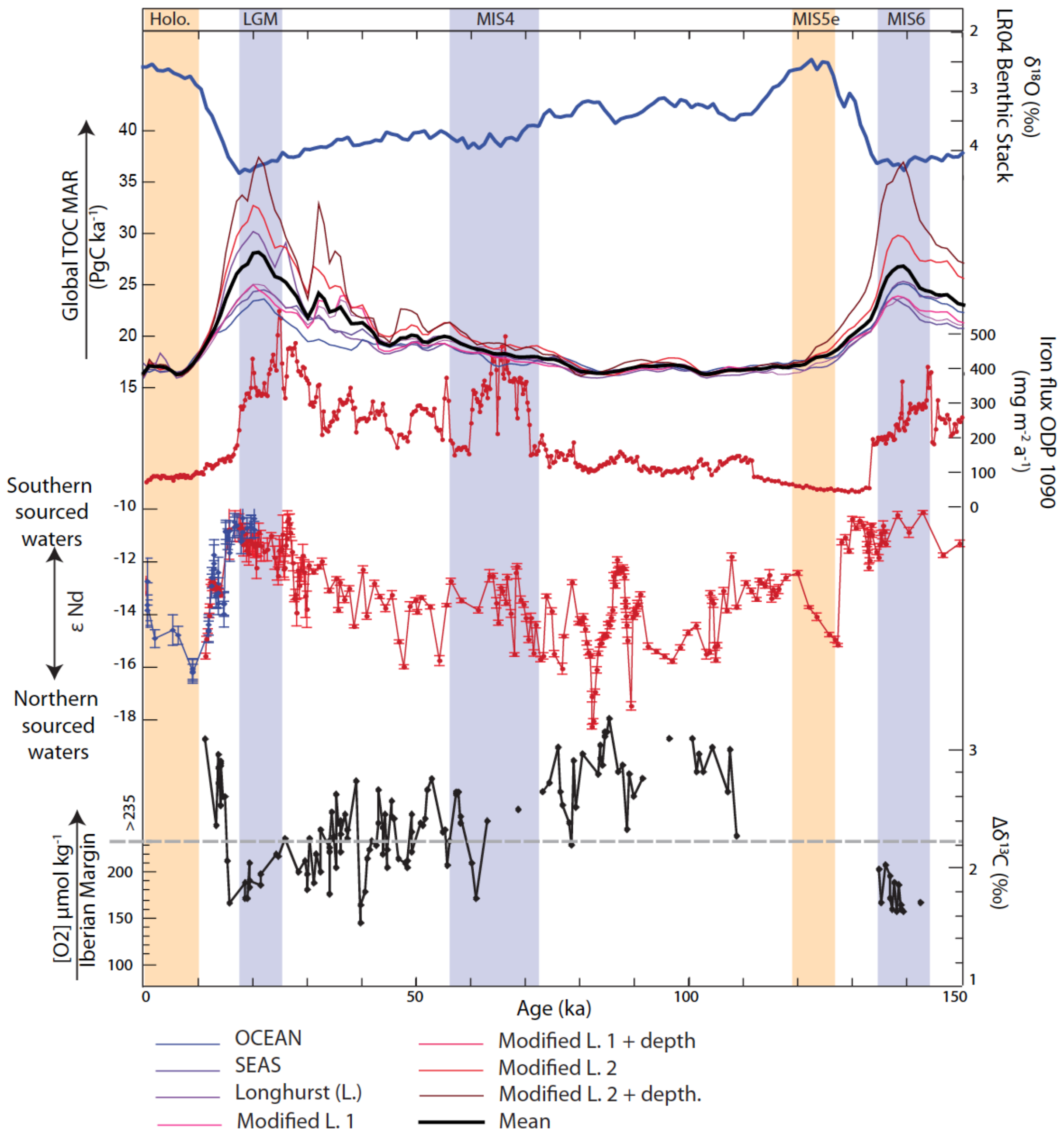

Supplementary Figure 5:
LR04 benthic foraminifera $\delta^{18}O$ stack; Reconstructed global TOC MAR in deep sea sediment (each line corresponds to a different province set scenario; the black thick line is the mean of all scenarios, Supplementary Data 1); Iron flux at ODP 1090 [5]; εNd in the northern Atlantic [6]; and reconstructed oxygenation in the northern Atlantic [7]. Grey horizontal bar on the bottom panel corresponds to the upper limit of oxygen content quantification for the method used in this study [7]. Yellow vertical bands correspond to interglacial periods (Holocene and Marine Isotopic Stage (MIS) 5e), blue vertical bands correspond to glacial condition (Last Glacial Maximum (LGM), MIS 4, and MIS6).

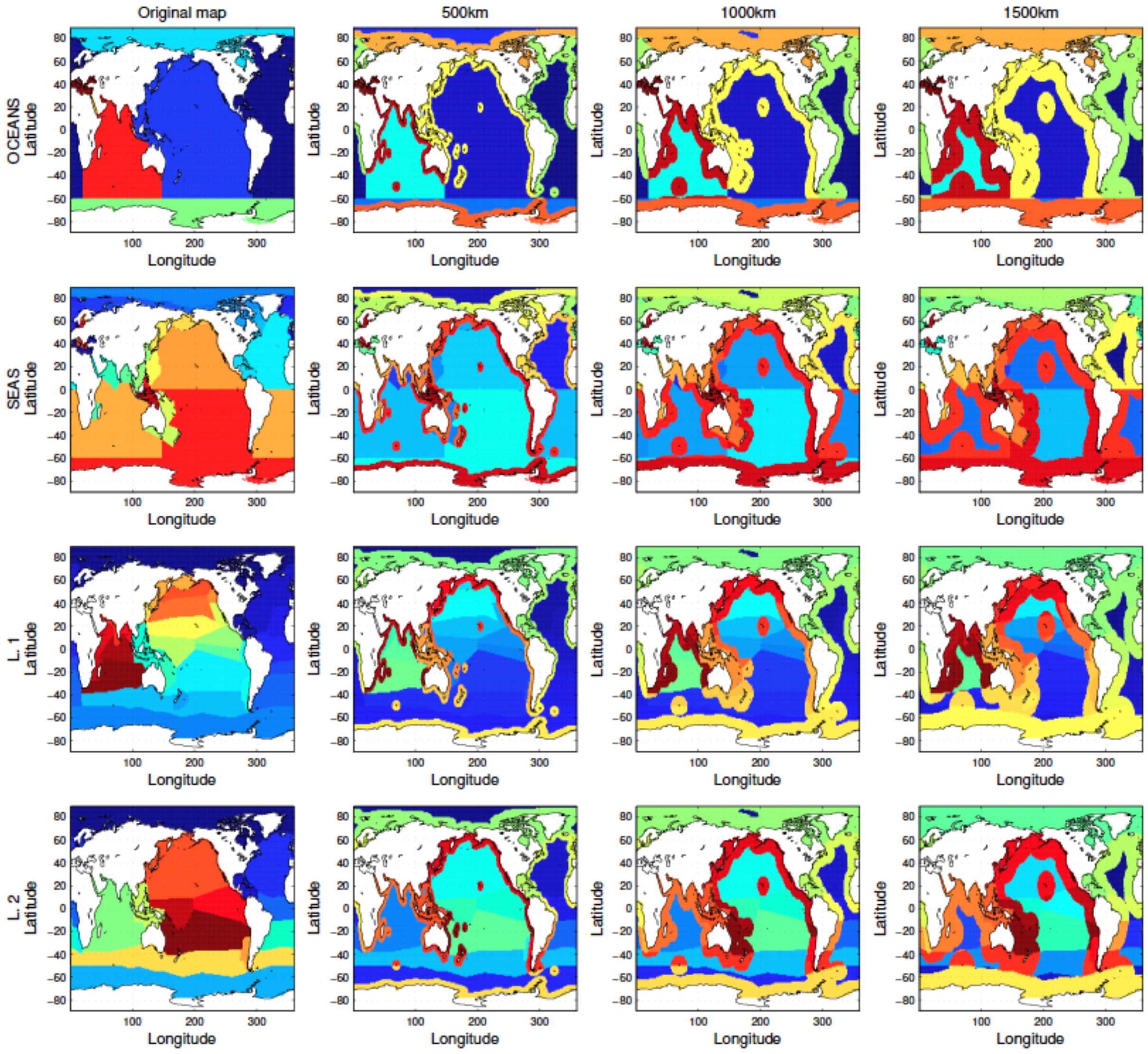

Supplementary Figure 6:
Province map a., b., d., and f. from Supplementary Fig. 2 were split into coastal and open ocean provinces, using a distance from the closest shoreline of 500 km, 1000 km and 1500 km. Each color corresponds to a single province. Global TOC MAR variations for split Oceans and Seas maps, are displayed in Supplementary Fig. 7.

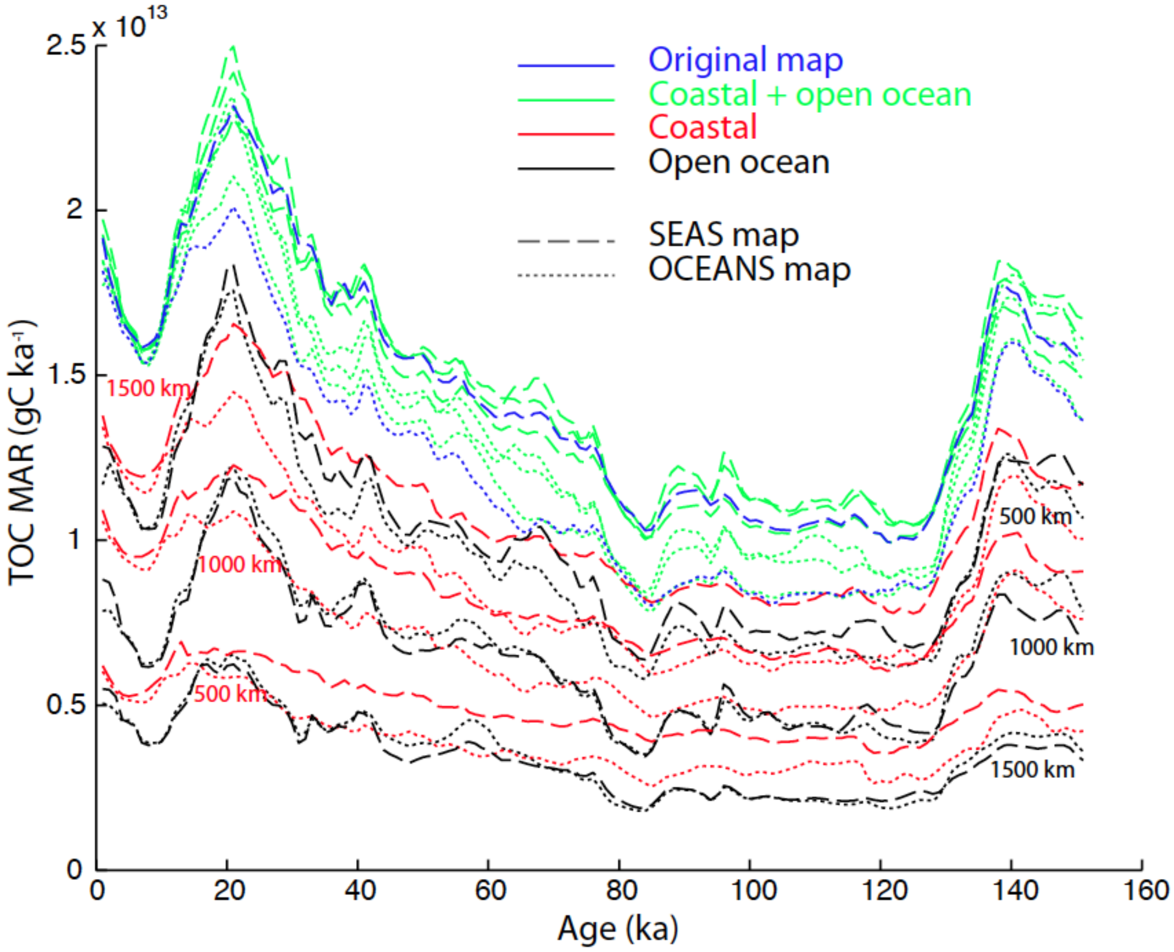

Supplementary Figure 7:
TOC MAR over the last 150 ka, for province map Ocean and Seas (bottom and top line of maps in Supplementary Fig. 6), including split provinces with distance from the coast. Blue lines correspond to the burial calculated with the original province maps. Black lines correspond to the sum of open ocean provinces, red lines to the sum of the coastal provinces. Green lines are the sum of open ocean and coastal region for each new province maps. Dotted lines are the scenarios based on the Ocean province maps, dashed lines are for Sea province maps.

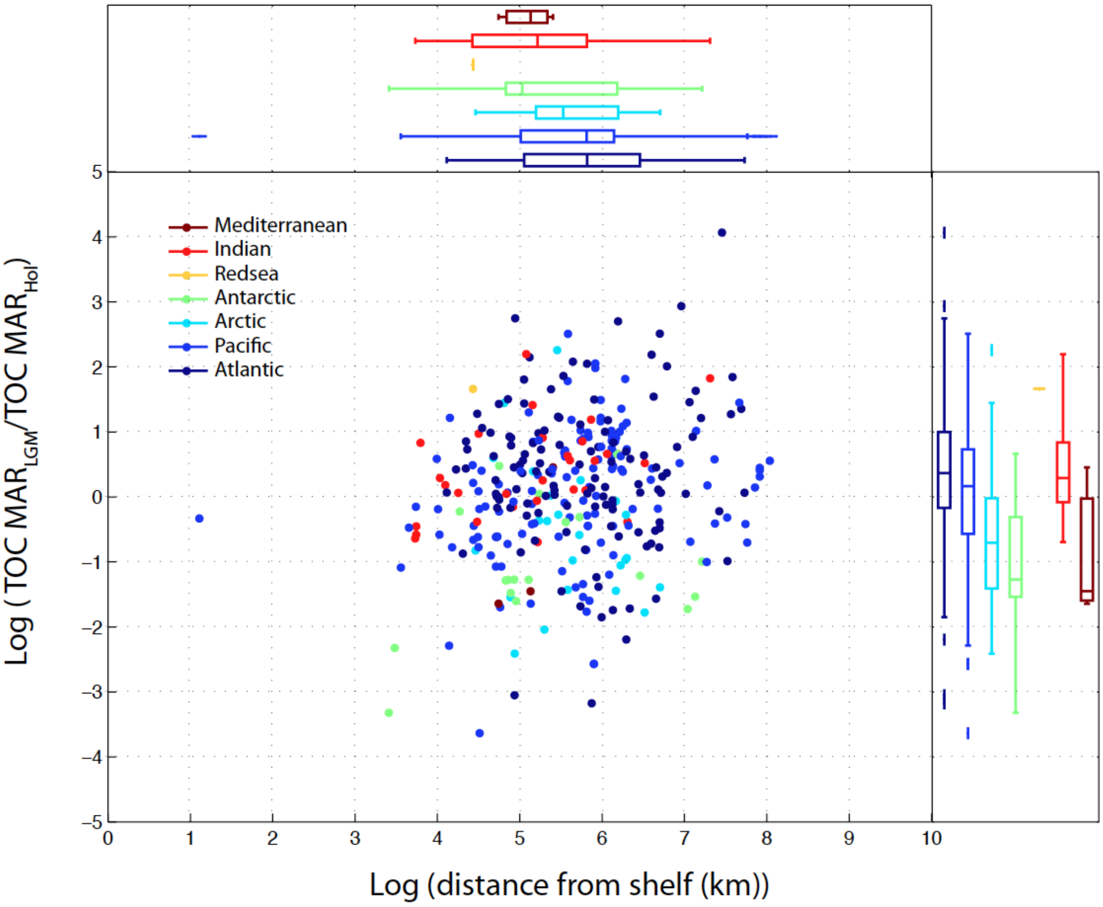

Supplementary Figure 8:

Log(TOC MAR_{LGM} /TOC MAR_{Hol}) of sedimentary records versus the distance from the closest 150 m isobath. The right and top panels display boxplot of log(LGM MAR/Holocene MAR) and log(distance from the shelf), separated by oceanic bassins. Box plot shows 0 % (bottom whiskers), 25 %, 50 % (median; middle line), 75 %, and 100 % (top whiskers) quantiles, as well as outliers (isolated ticks).

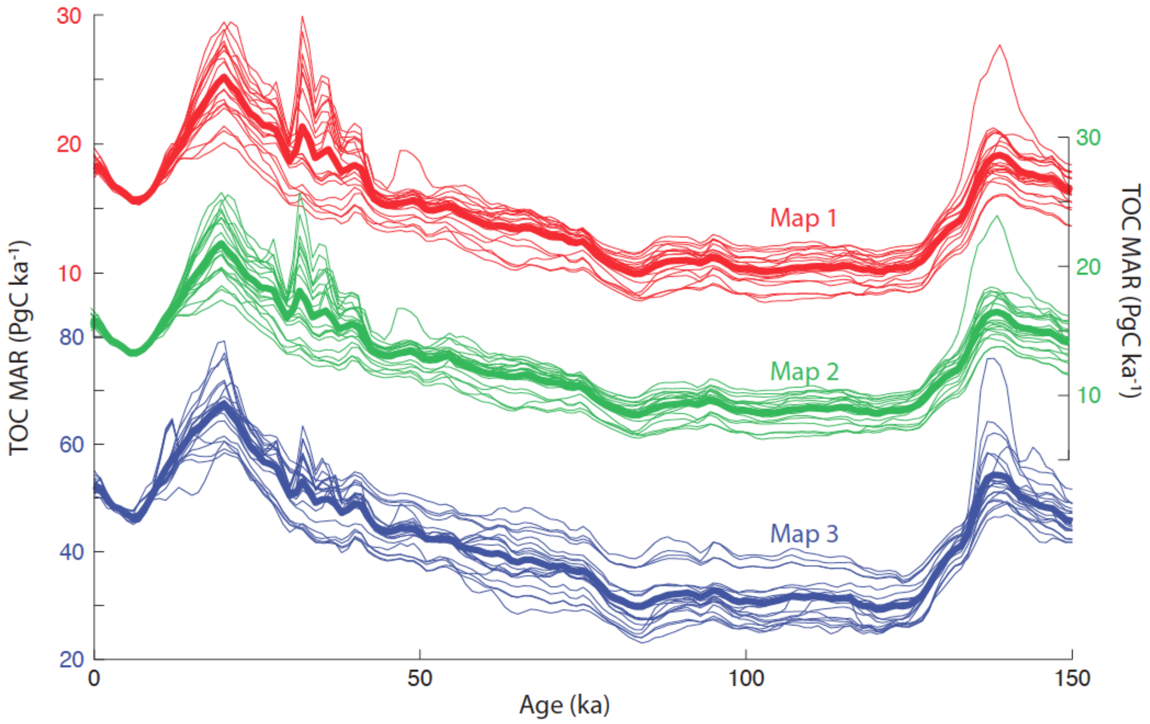

Supplementary Figure 9:
Global TOC MAR using different modern TOC MAR maps. Map 1 is for the modern map used in this study [1], Map 2 corresponds to an updated TOC content map, and only the Jahnke bulk MAR map [8]; and Map 3 corresponds to the global map of modeled organic carbon flux to the seafloor from Ref. [9] (see Methods). Each thin line corresponds to one province map scenario, thick lines correspond to the mean of the different scenarios, for each modern burial map. Note that this figure presents results for the province map shown in Supplementary Fig. 2 and 6.

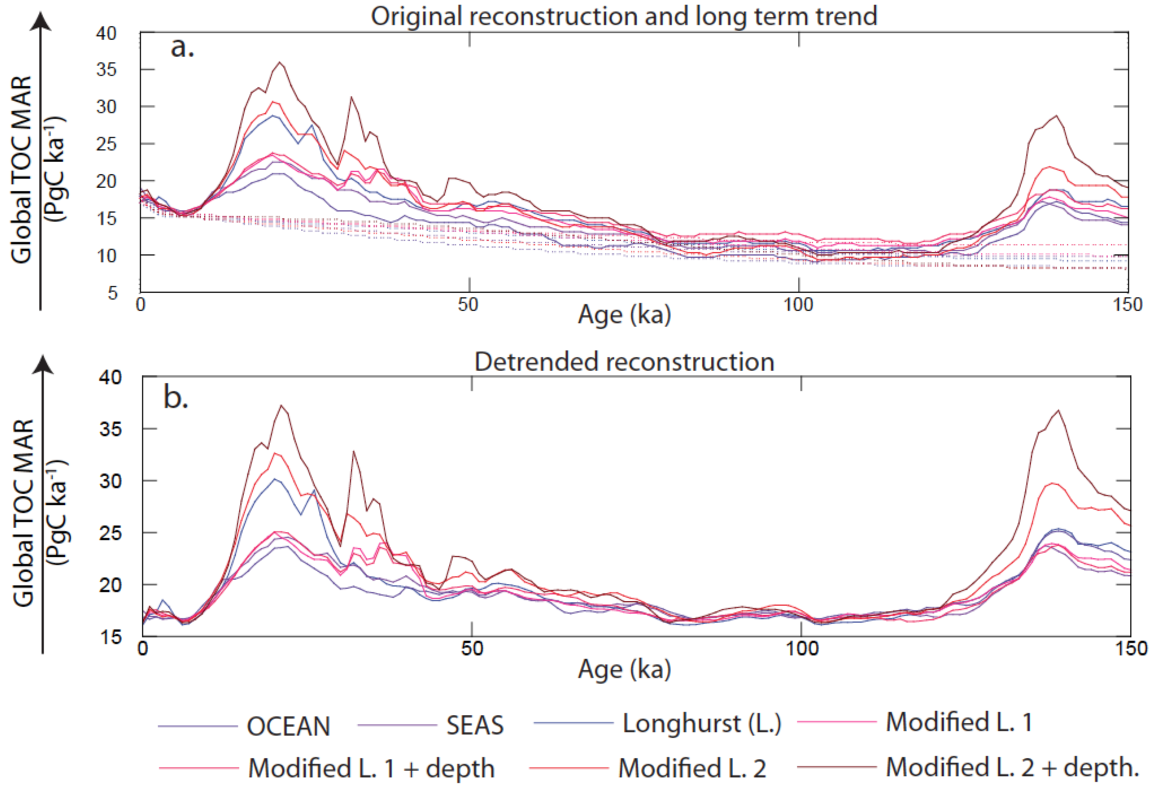

Supplementary Figure 10:
a. Original reconstructions (lines) and long-term trends (dotted lines) in TOC MAR over the past 150 ka for each province map scenarios. b. Detrended reconstructions (Supplementary data 1).

Supplementary references:

1. Dunne JP, Sarmiento JL, Gnanadesikan A. A synthesis of global particle export from the surface ocean and cycling through the ocean interior and on the seafloor. *Global Biogeochem Cycles* **21**, GB4006 (2007).

2. International Hydrographic Organization. Limits of Oceans and Seas. *International Hydrographic Organization, Special Publication* **23**, (1953).

3. Longhurst A. Seasonal cycles of pelagic production and consumption. *Prog Oceanogr* **36**, 77-167 (1995).

4. Reygondeau G, Longhurst A, Martinez E, Beaugrand G, Antoine D, Maury O. Dynamic biogeochemical provinces in the global ocean. *Global Biogeochem Cycles* **27**, 1046-1058 (2013).

5. Martinez-Garcia A, Rosell-Mele A, Jaccard SL, Geibert W, Sigman DM, Haug GH. Southern Ocean dust-climate coupling over the past four million years. *Nature* **476**, 312-315 (2011).

6. Bohm E*, et al.* Strong and deep Atlantic meridional overturning circulation during the last glacial cycle. *Nature* **517**, 73-76 (2015).

7. Hoogakker BAA, Elderfield H, Schmiedl G, McCave IN, Rickaby REM. Glacial-interglacial changes in bottom-water oxygen content on the Portuguese margin. *Nature Geosci* **8**, 40-43 (2015).

8. Jahnke RA. The global ocean flux of particulate organic carbon: Areal distribution and magnitude. *Global Biogeochem Cycles* **10**, 71-88 (1996).

9. Dunne JP, Hales B, Toggweiler JR. Global calcite cycling constrained by sediment preservation controls. *Global Biogeochem Cycles* **26**, GB3023 (2012).
